# Theoretical Studies and Implementation on the Temporary Data Storage Method for Cone Penetration Test

**DOI:** 10.3390/s21020575

**Published:** 2021-01-15

**Authors:** Yanming Li, Yuheng Shen, Xiaoquan Wang, Sifeng Li, Tonglu Li, Quanli Zhao

**Affiliations:** 1 School of Electronic and Control Engineering, Chang’an University, Xi’an 710064, China; syh19960805@163.com (Y.S.); wangxiaoquan9999@163.com (X.W.); lisifeng970830@163.com (S.L.); 2 College of Geology Engineering and Geomatics, Chang’an University, Xi’an 710054, China; dcdgx08@chd.edu.cn (T.L.); zhaoquanli@chd.edu.cn (Q.Z.)

**Keywords:** CPT, cableless, data accuracy, underground sensor

## Abstract

The traditional cone penetration test system uses cable to transmit data; as the probe goes deeper into the ground, the length of the cable will become longer. This makes the installation of the test equipment more complicated, and excessively long cables cause signal distortion and seriously affect data accuracy. To simplify the experimental equipment and improve the accuracy of data acquisition, a cableless cone penetration test system is proposed. The improved system uses an SD card to store the experimental data, as opposed to using cables for communication which, often lead to the distortion of signals caused by long-distance communication and data loss caused by accidental cable breaks. Therefore, the accuracy of the collected data is higher, and the experimental device is simplified. To evaluate the applicability and efficiency of our design, we have carried out exploration experiments with the sensor system proposed in this paper. The test results show that the experimental data collected by the new system are basically consistent with the data collected by traditional cable CPT equipment, and the accuracy of the collected data is higher. It is more reliable and accurate to analyze the comprehensive mechanical properties of the soil layers with the data collected by the new system.

## 1. Introduction

**Figure 1:**
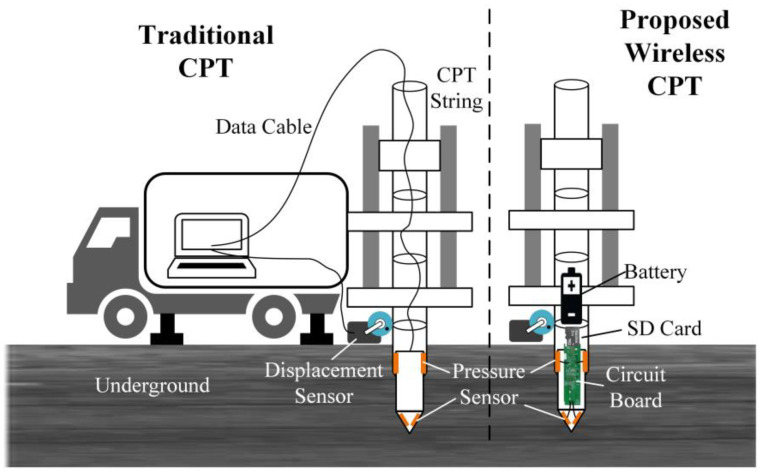
Cone penetration vehicle.

The cone penetration test (CPT) involves pushing a probe with sensors into the soil at a constant speed through quasi-static force, and measures the stress that the cone tip and the side wall of the probe receive during the process. The soil property can be inferred by analyzing the stress of the probe at different depths. Because of the low cost and high reliability, CPT has been widely used in the field of geological exploration, such as in underground soil testing [1], building foundation monitoring, groundwater monitoring [2,3,4,5,6,7], and assessments of effective stress state in soils [8]. Typically, the sensing device for underground exploration is a probe with a cone-shaped tip, and various sensors can be installed inside the probe [9]. During the cone penetration test, how to save the data collected by sensors is a key issue [10]. At present, most systems use a data cable to transmit the data collected by sensors to the receiving equipment on the ground in real-time, and these data are then stored by the receiving equipment. During the experiment, the data cable needs to be passed through a long and narrow steel pipe so that the data collected by sensors can be transmitted above ground. In fact, it is a widely used technique using cables to transmit the sensor signal. In the experiment, the probe is pushed into the ground with a cone penetration vehicle [11]; the experimental device is shown in the left half of Figure 1. This machine drives the probe through soil layers with a set of steel rods called CPT strings. By applying a controllable force or vibration to the CPT strings on the ground, the probe will gradually be pushed deeper into the ground. The movement of the CPT string underground causes the roller of the displacement sensor to rotate. The displacement sensor will record the depth of the probe under the ground at different times. The CPT strings are cylindrical steel rods that can be screwed together so that the probe can go as deep as desired. In practice, the data cable has to be threaded through all the rods before drilling. As the probe goes deeper into the ground, the signal collected by the pressure sensor will be transmitted to the receiving equipment through the cable. The receiving equipment has the functions of signal amplification, analog-to-digital conversion and data processing. After receiving the data, the receiving equipment will convert the signal into a digital quantity and store it in the TXT file. At present, KE-2103 is the most widely used traditional CPT system [12]. KE-2103 adopts an 8-bit ADC to collect the sensor’s signals; the measurement accuracy of the cone tip pressure is 0.01 MPa, and the accuracy of the side wall pressure is 0.1 kPa. The traditional CPT system is more time-consuming and inconvenient. Moreover, the use of cables for communication will be limited by the length of the cable—the longer the cable is, the more noise will appear in the transmission process of the signal, and the easier the signal distortion will be. As analyzed by T. Meyer, noise has a great influence on the work of the sensor [13], and the maximum exploration depth of the probe will be limited due to the noise signal and distortion.

Therefore, the experimenters hope to develop a cableless system that can transmit data from a probe deep underground to receiving equipment on the ground. At present, various cableless technologies exist that transmit sensory data from underground probes to the ground. Examples include ultrasonic, underground radio and magnetic induction [14,15,16,17,18]. However, due to the influence of humidity, soil moisture and temperature [19,20], these cableless communication technologies are not reliable in the practical application of the cone penetration test. The data received by the receiving equipment are noisy, which affects the analysis of the soil properties by experimenters. Moreover, the distance of signal transmission is not as long as the cable mode, and the purchase cost of the equipment is higher.

In fact, these technologies are designed based on the idea of transmitting sensory data to the receiving equipment in real-time. However, CPT has no requirements on the real-time performance of sensory data. We can temporarily store the data collected by sensors in a data storage device in the probe. After an exploration, the probe needs to be reinserted into the ground, and then the experimental data in the data storage device are copied to data-receiving equipment, such as a computer. Based on this idea, we designed the cableless CPT system proposed in this paper, which integrates sensors, amplifier, MCU and SD card compactly. The circuit board can be embedded in the rod. The SD card mounted on the circuit board is used to temporarily store the experimental data collected by the sensors. Furthermore, the working state of the system is controlled by the MCU, and the probe can work completely autonomously after going down to the ground. After one exploration, one needs to disassemble the probe rod and remove the SD card on the circuit board, and then read the experimental data stored in the SD card with a card reader. The proposed system avoids many problems caused by using long cables. The structure of the new device is shown in the right half of Figure 1. The data cable is omitted in the experimental equipment. The circuit board and battery are installed inside the probe rod. The dimensions of the probe and the rod are consistent with those used in the traditional equipment. To verify the feasibility of our idea, we have carried out the physical production and field exploration tests. The test results showed that the experimental data collected by the new system are basically consistent with the data collected by traditional cable CPT equipment, and the accuracy of the collected data is higher. It is more reliable and accurate to analyze the comprehensive mechanical properties of the soil layers with the data collected by the new system.

## 2. System Design

**Figure 2:**
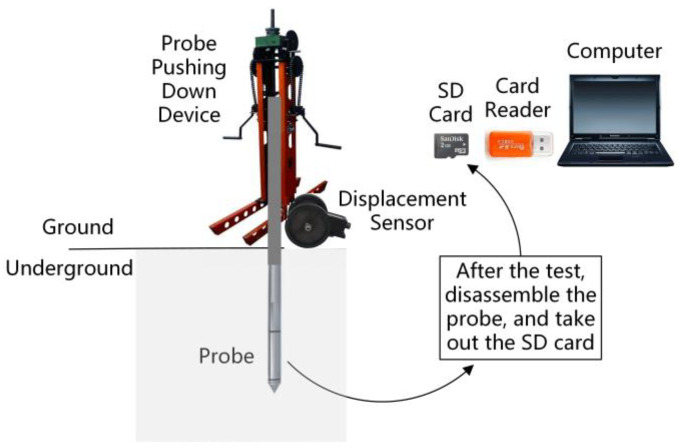
The diagram of the signal acquisition process of the proposed sensor system.

The whole set of the CPT system includes the probe, pressure sensor, displacement sensor, data acquisition circuit board and control program. Figure 2 shows the signal acquisition process of the proposed sensor system. The pressure sensor is embedded in the probe, and the data acquisition circuit board is used to amplify the analog signal and store the experimental data, and is installed in the probe tail pipe. In the field experiment, we need to connect the data acquisition circuit board and the displacement sensor with a data cable first, and then press the synchronization button on the displacement sensor. The displacement sensor and the pressure sensor will start timing from 00:00:00 at the same time. Finally, the probe pushing-down device is used to push the probe into the ground.

In the process of pushing the probe into the ground, pressure sensors installed in the cone tip and probe side wall convert the pressure received by the probe into an electrical signal, which is amplified by an amplifier circuit and then input into an AD converter. Finally, the experimental data are stored in the SD card by the MCU. The data in the SD card can be imported into the computer by using a card reader. The data analysis software of the system will calculate the size of the resistance of the probe received in different depths of soil layers according to the experimental data, and provide a basis for inferring the soil properties. In addition, a lithium battery is fixed in the probe tail pipe to power the system. The improved system does not need to transmit the experimental data to the receiving equipment on the ground through cables. After the probe is pushed into the ground, it is controlled by the control program and can work completely autonomously. Hence the negative impact of using cables for communication is avoided. Furthermore, the proposed system adopts a 12-bit ADC; compared with the 8-bit ADC in KE-2103, the new system has a higher sampling accuracy.

## 3. Pressure Sensor Design

**Figure 3:**
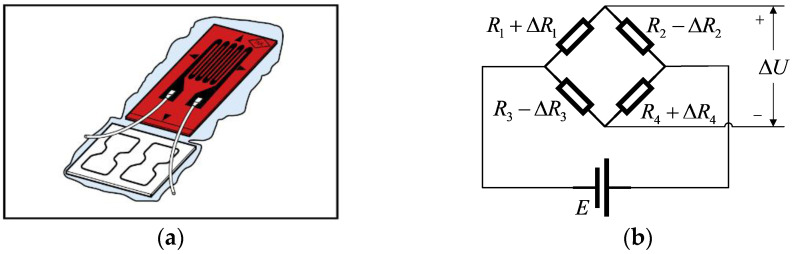
Structure and application of a strain gauge. (**a**) Strain gauge structure diagram. (**b**) Full bridge measuring circuit.

The probe used in the cone penetration test is a cylinder made of metal material with a cone-shaped tip. The probe can be pushed into the ground at a constant speed by a hydraulic machine. In order to obtain the stress of the soil layer encountered by the probe during the process of pushing, pressure sensors should be installed inside the cone tip and the side wall of the probe. The pressure sensor is composed of four metal foil resistance strain gauges. Inside the strain gauge is a very thin and long metal wire, which is wound into a rectangle and pasted between the plastic protective film. The structure of the strain gauge is shown in Figure 3a. When the strain gauge is bent by external forces, the length of the inner metal wire will become longer, and the resistance value *R* of the metal wire is proportional to the length *L*. The increase in the length will lead to an increase in the resistance of the strain gauge [21,22,23].

In order to select suitable resistance strain gauges to make pressure sensors, we referred to the strain gauge products of Vishay Precision Group, Inc. [24] and Tokyo Measuring Instruments Lab [25]. Several universal strain gauge product series are listed in Table 1. According to the characteristics of different types of products and combined with the requirements of the cone penetration test, we have chosen the BF series strain gauge, which is more suitable for high-precision pressure sensors to use.

Four strain gauges have to be connected into a bridge circuit as shown in Figure 3b. When the cone tip is subjected to resistance, the cone tip will produce a slight deformation and cause the internal strain gauges to bend, resulting in a change in the resistance value of the strain gauges and the imbalance of the bridge. When a voltage-stabilized source or a constant current source is added to the bridge circuit, the change in resistance is converted into an output voltage Δ*U* [26].

Two sets of the same pressure sensors are installed in the cone tip and the side wall of the probe. When the strain gauge is not subjected to external pressure, the resistance values of the strain gauge are *R*_1_, *R*_2_, *R*_3_ and *R*_4_, according to the related physical laws of the full bridge circuit [27], the relationship between Δ*U* and the resistance of strain gauges in Figure 3b can be expressed as:

(1) $$\Delta U=E\left(\frac{R_{1}+\Delta R_{1}}{R_{1}+\Delta R_{1}+R_{2}-\Delta R_{2}}-\frac{R_{3}-\Delta R_{3}}{R_{3}-\Delta R_{3}+R_{4}+\Delta R_{4}}\right)$$

The cableless CPT system proposed in this paper uses the built-in 12-bit ADC of the single-chip microcomputer to convert the analog voltage signal Δ*U* into a four-digit decimal number, and stores these numbers in the SD card. Users can apply Equation (2) to convert the numbers stored in the SD card into the output voltage Δ*U* generated when the bridge circuit is unbalanced:

(2) $$\Delta U=\left(X\cdot \frac{3.3}{4096}\right)/G$$

where *X* is the value stored in the SD card, *G* is the magnification of the operational amplifier AD620, and Δ*U* is the output voltage of the full bridge measurement circuit.

In order to establish the corresponding relationship between the resistance of the strain gauges and the force exerted on the probe, this paper takes the cone tip as an example. Through experimental calibration, it is observed that the change in the resistance when the strain gauge is deformed is proportional to the force exerted on the cone tip. The corresponding relationship can be expressed by Equation (3):

(3) ΔR=αF

By substituting Δ*R* in Equation (3) into Equation (1), the relationship between the force on the cone tip and the output voltage of the full bridge circuit can be obtained. Since the output voltage Δ*U* can be obtained from Equation (2), the corresponding relationship between the value *X* stored in the SD card and the force *F* exerted on the cone tip can finally be derived through the following formula:

(4) $$\Delta U=\left(X\cdot \frac{3.3}{4096}\right)/G=E\left(\frac{R_{1}+\alpha_{1}F_{1}}{R_{1}+\alpha_{1}F+R_{2}-\alpha_{2}F_{2}}-\frac{R_{3}-\alpha_{3}F_{3}}{R_{3}-\alpha_{3}F_{3}+R_{4}+\alpha_{4}F_{4}}\right)$$

Because the four strain gauges are uniformly pasted inside the probe cone tip, and the force on the cone tip is symmetrical when the probe is pushed into the soil layer, it is reasonable to consider that *F*_1_ = *F*_2_ = *F*_3_ = *F*_4_, and the four strain gauges are selected from the same type of products with no difference from each other. We can also consider that *α*_1_ = *α*_2_ = *α*_3_ = *α*_4_, and *R*_1_ = *R*_2_ = *R*_3_ = *R*_4_. After substituting these conditions, Equation (4) can be simplified as follows:

(5) $$\left(X\cdot \frac{3.3}{4096}\right)/G=E\cdot \frac{\alpha F}{2R}$$

Equation (5) can be modified as:

(6) $$F=\frac{3.3XR}{2048\alpha GE}$$

When analyzing the experimental data, the data in the SD card can be converted into the force exerted on the probe cone tip and side wall by Equation (6), and then the soil property can be inferred by analyzing the force.

## 4. Analog Voltage Amplification and Acquisition Circuit

**Figure 4:**
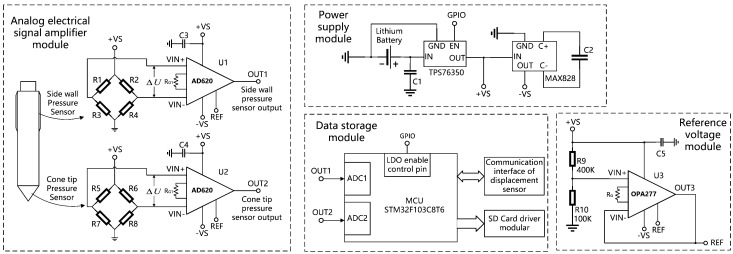
Diagram of analog voltage amplification and acquisition circuit.

Figure 4 shows the diagram of the analog voltage amplification and acquisition circuit of the proposed cableless CPT system. This circuit is used to amplify the output voltage Δ*U* of the bridge circuit of the pressure sensor, and then convert the amplified analog signal into a digital value through an AD converter, and finally the digital value will be stored in the SD card by a single-chip microcomputer. The circuit board is powered by the power supply module, and the working state of the power supply module can be controlled by the MCU. When the MCU does not execute the data acquisition instruction, the power supply to the pressure sensors can be turned off to reduce system power consumption.

In the analog electrical signal amplifier module, two precision instrumentation amplifiers AD620 are used to amplify the output voltages of two bridge circuits in the cone tip and the side wall, respectively, and the amplified voltage signals are respectively input into the input pins of the two 12-bit AD converters of the MCU. In order to prevent the bridge circuit from outputting negative voltage, which will make the AD620 output negative voltage and cause the AD converters in the MCU to be unable to process the input signal, it is necessary to provide a reference voltage for the amplifiers through the reference voltage module. The reference voltage module uses OPA277 to form a buffer circuit, which provides 1V reference voltage for two AD620 chips, so that even if the bridge circuit outputs a negative voltage, it will not cause the AD620 to output a negative voltage.

The data storage module is used to convert the 12-bit binary number output by the AD converter into a four-digit decimal number, and store these numbers in an SD card. The core component of this module is a STM32F103xx ARM-based 32-bit MCU, which controls the whole system by running the control program. In addition, the module can also control the enable pin of the power supply module through an IO pin, so that the MCU can control whether to supply power to the pressure sensors, and communicate with the displacement sensor to realize the function of synchronously starting the timing of the two devices.

## 5. Control Program

According to the requirements of the cone penetration test, the MCU realized the functions of analog signal converting, SD card driving, data storage, system time, and time synchronization with displacement sensor, etc.

**Figure 5:**
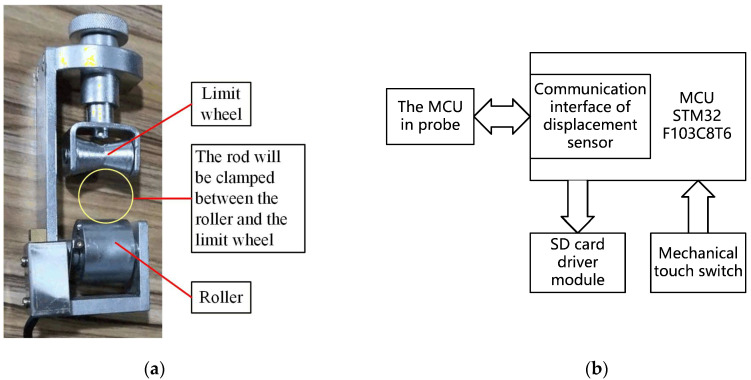
Displacement sensor: (**a**) physical drawing of displacement sensor; (**b**) block diagram of displacement sensor system.

In the process of pushing the probe into the ground, the MCU records a group of experimental data every second. The SD card can save the force data of the probe cone tip and side wall at each sampling time, but it cannot record the depth of the probe under the ground at that time. In order to get the depth of the probe at each sampling time, the displacement sensor shown in Figure 5 is needed.

The displacement sensor is adopted to record the depth of the probe, and its physical diagram is shown in Figure 5a. The roller and limit wheel of the displacement sensor must hold the rod tightly, and the displacement sensor should be fixed on the base of the probe pushing-down device. In this way, when the rods move downward, they can drive the roller to rotate. By counting the closing times of the switch, we can know how many times the roller has turned, and calculate the depth of the probe that has been pushed into the ground. The core component of the displacement sensor is a cylindrical roller. There is a striker on the bottom of the cylinder. Every time the roller rotates, the striker will touch the switch once and close the switch instantaneously. By counting the number of switch closures, we can know how many turns the roller has turned, and then calculate the depth of the probe that has been pushed into the ground. Before the probe is pushed into the ground, the experimenter needs to use a data cable to make the displacement sensor and the pressure sensor obtain the same timing clock, and then the two devices will start timing at the same time. The displacement sensor records the accumulated rotation number of the roller, and stores these data in its SD card every other second. In addition, the data acquisition circuit board will record the force data of the probe cone tip and the side wall in the SD card every other second too. The user can process the data of two SD cards in the displacement sensor and the probe through the supporting software, and get the size of the force that the probe receives at different depths and analyze the properties of the soil layer.

The purpose of collecting a set of data every other second under the control of MCU is to reduce the power consumption of the system. When the data acquisition and storage program is not executed, the MCU will enter the sleep mode to reduce power consumption [28], and wait for the timer to count for one second before executing the next data acquisition and storage program. The first step of the data acquisition and storage program is to enable the LDO chip to output + 5 V voltage to drive the amplifiers AD620 and provide working voltage for pressure sensors. The second step is to control the AD converter in the MCU to collect analog electrical signals ten times, and convert them into digital values, then take the average of these ten converted digital values, and store the results in the on-chip memory of MCU. The third step is to turn off the LDO chip and stop the power supply to the amplifiers and pressure sensors to reduce system power consumption. The last step is to write the results stored in the on-chip memory to the SD card through the FATFS file management system [29,30], and write the current system time to the SD card at the same time, so that the data read by the user from the SD card has both the digital values converted by the AD converter and the recording time of the digital values.

**Figure 6:**
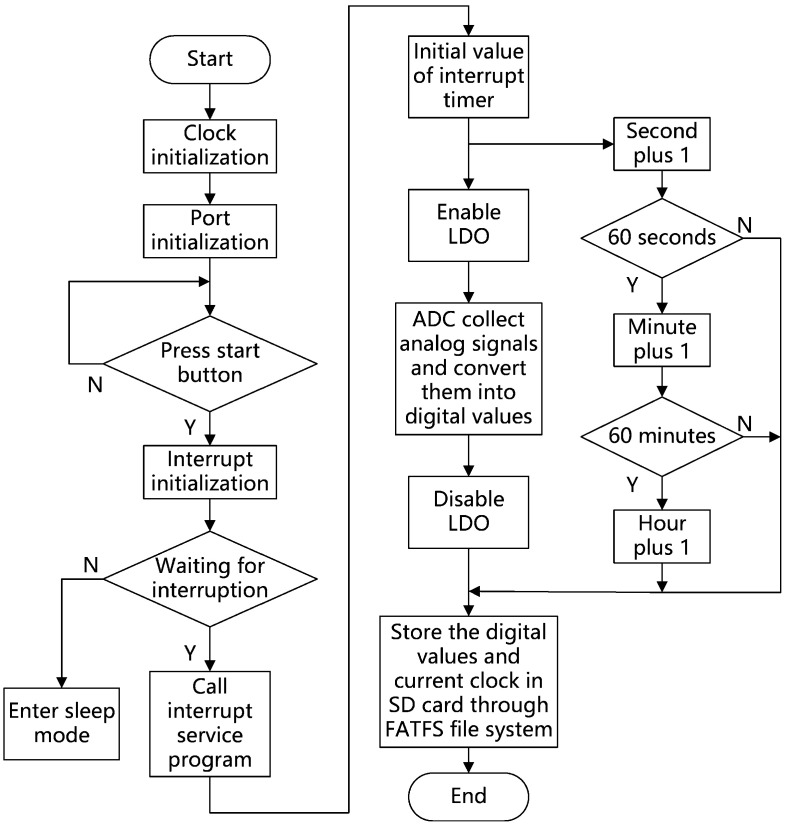
Program flow chart.

According to the function division, the program flow chart of MCU is shown in Figure 6:

## 6. Experimental Results and Discussions

**Figure 7:**
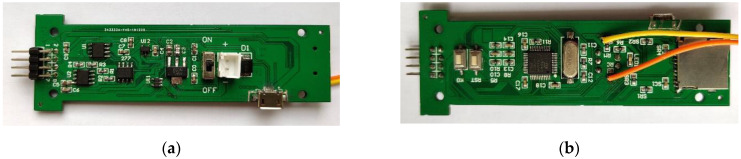
Analog voltage amplification and acquisition circuit: (**a**) front of the circuit board; (**b**) back of the circuit board.

The probe used in the cableless CPT system proposed in this paper is a cylinder made of metal material with a cone-shaped tip. The bottom area of the probe is 15 cm^2^, the top angle of the cone is 60°, and the cone tip can withstand a maximum force of 6300 N [31]. In the cone penetration test, a lot of probe rods will be installed behind the probe, and the probe pushing-down device will press the probe rod down at a constant speed, thereby driving the probe to continuously push down into the ground. In order to install the circuit board and battery in the rod, we made a special probe rod. The dimensions of the probe rod are the same as other probe rods; its inner diameter is 35 mm, and the wall thickness of the probe rod is 9 mm, which is a standard probe rod. Inside the special probe rod, there is a clamping mechanism for fixing the circuit board and battery. In order to match the clamping mechanism of the circuit board, and to be able to install the circuit board inside the probe rod, we designed the analog voltage amplification and acquisition circuit board into a thin and long T-shaped structure. The physical structure of the circuit board is shown in Figure 7. The size of the circuit board is 90 × 25 mm, which meets the design requirements of miniaturization of the circuit board, so that the circuit board can be installed inside the probe rod.

**Figure 8:**
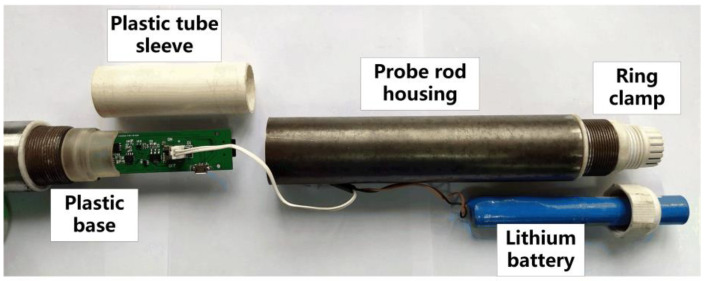
Probe rod assembly diagram.

The clamping mechanism for fixing the circuit board and the battery in the probe rod is shown in Figure 8.

The plastic base is used to clamp the T-shaped ears which are on the circuit board, and a female header is installed in the base to connect with the pin header on the circuit board. There is a circle of threads outside the plastic base and the inner wall of the probe rod, so the plastic base can be fastened in the probe rod. After inserting the circuit board into the plastic base, the plastic tube sleeve needs to be fastened to the base, which can cover the whole circuit board and protect it. Behind the circuit board is a cylindrical lithium battery. We use a ring clamp to fix the battery. The ring clamp is glued to the end of the probe rod. The battery can be fixed by inserting the battery into the ring clamp and then tightening the fixing nut. After the circuit board and battery are fixed, the special probe rod can be installed on the tail of the probe, and the cone penetration test can be performed. The special probe rod has the same dimensions as the standard rod, and the connection and disassembly methods are also the same as the standard rod. This allows the improved probe to still be able to be pushed into the soil layer through the traditional probe pushing-down device. There is no need to replace the pushing device for the new probe, which reduces the replacement cost of the new device and is conducive to the popularization of the new probe. Furthermore, traditional probes use cables to transmit experimental data; limited by the length of the cable, the probe can be pushed into the ground for 30 m at most. If the depth exceeds 30 m, the receiving equipment will fail to receive valid experimental data. However, the new probe proposed in this paper will not be subject to such limitations. Within the allowable range of the pressure provided by the probe pushing-down device, the maximum exploration depth of the probe is not limited by the cable, which will greatly improve the exploration capability of the CPT equipment.

The cableless CPT system introduced in this paper should be used according to the following steps:

(1) Open the sealed protective cover of the first probe rod behind the probe, and insert the SD card into the SD card slot at the end of the circuit board;
(2) Connect the circuit board in the probe rod and displacement sensor with a data cable, and then press the clock synchronization button on the displacement sensor. After the button is pressed, the data cable can be removed, and the sealed protective cover of the probe rod can be screwed back;
(3) Level the test site with a shovel and other tools, install the hydraulic machine in place, ensure that the probe is vertically pushed into the soil layer, fix the displacement sensor at the bottom of the hydraulic machine, and fit the probe into the fixed seat of the displacement sensor;
(4) Push the probe into the soil layer at a constant speed by the probe pushing-down device. When the length of the probe rod is not enough, experimenters need to manually connect more rods until the required exploration depth is reached;
(5) After one test, the probe rod should be pulled out immediately. Disassemble the first section of the probe rod behind the probe, and remove the SD card in the tail pipe of the probe, then copy the experimental data in the SD card to computer through a card reader.

**Figure 9:**
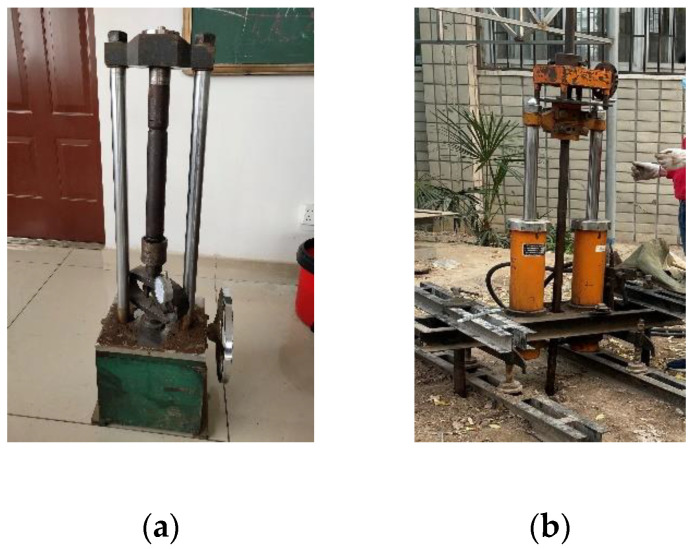
Probe calibration and field exploration: (**a**) probe calibration frame; (**b**) probe pushing-down device.

The cableless CPT system proposed in this paper was firstly calibrated and simulated in the laboratory after the sample was produced in December 2019. Under the condition that the indoor environment is basically constant, the probe calibration frame as shown in Figure 9a is used to apply pressure to the probe, so that the force on the probe tip can gradually increase from 0 N to the maximum force that the probe can withstand. After a pressurization process, we read the experimental data stored in the SD card.

Some experimental data are shown in Table 2. The data stored in the SD cards of the probe and displacement sensor are merged into Table 2. The data in the two SD cards have the same acquisition time. The column ‘data1’ is the data collected by the probe cone tip pressure sensors. The column ‘data2’ is the data collected by the side wall pressure sensors. The column ‘round’ is the accumulated rotation number of the displacement sensor. After the two devices are synchronized, the displacement and pressure data are collected at the same collection rate. After the experiment, the displacement and pressure data can be easily correlated by time alignment. It is found that the experimental data gradually increase from 20 to 4000, which means that when the probe is suspended, the corresponding value is 20; when the probe is subjected to the maximum pressure, the corresponding value is 4000. The test result meets the design requirements.

**Figure 10:**
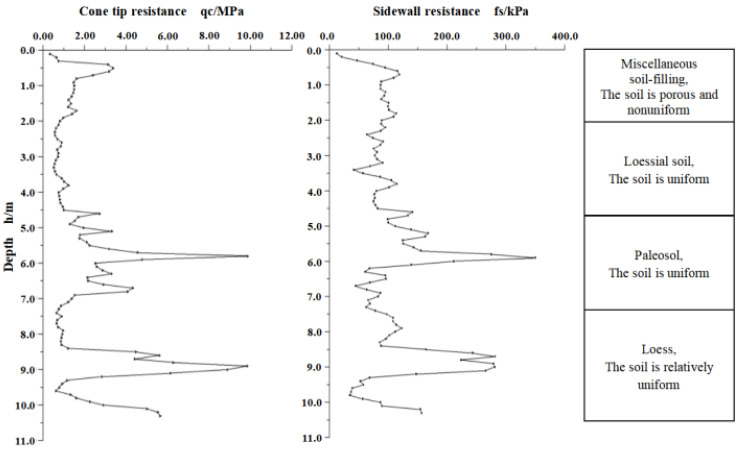
The curve diagram of the stress that the probe receives.

After the indoor simulation test was successful, a field exploration test with the new system was carried out, and the probe pushing-down device is shown in Figure 9b. The device is powered by a diesel engine and applies vertical downward pressure to the probe and the probe rods through a hydraulic press. The system was set to store a set of experimental data every other second, and the probe was pushed into the soil layer to the depth of 20 m. The process of the experiment involved three experimenters and 6 h in total. If we use a traditional cable system, we need five experimenters and 12 h. The extra experimenters and time are mainly spent on threading the cable through the rods. By comparison, the proposed system significant decreased the cost of experimenters and the time of CPT. After the test, we took out the SD card from the probe, and used a card reader to copy the TXT file in the SD card to the PC. There were about 1000 sets of experimental data stored in the file. By running the supporting data analysis software of this system on a PC, the experimental data in the form of text in the TXT file can be drawn into a curve diagram of the stresses that the probe tip and side wall receive at different depths. The curve diagram is automatically drawn by the software, and one such example is shown in Figure 10. According to the curve diagram, researchers can infer the comprehensive mechanical properties of the soil layers at different depths underground [32]. By analyzing the data collected by the new system, we saw that the measurement accuracy was up to 0.02 kPa. However, if we use the traditional cable CPT system KE-2103 to collect the data, the measurement accuracy of the probe cone tip pressure is 0.01MPa, and the measurement accuracy of the side wall pressure is 0.1 kPa. So, we can get a more accurate conclusion by using the data collected by the new equipment to analyze the soil layer.

## 7. Conclusions

Based on the traditional equipment, this system converts and improves the traditional method of transmitting the sensory data by cable to the method of temporarily storing data in SD cards, which performs fully autonomous work after the probe is down in the ground, and avoids many problems caused by using long cables for communication. Furthermore, the control chip used in the system has a 12-bit AD converter, which is more accurate than the traditional CPT system, so the experimental data collected by the improved system have a higher accuracy and reliability. In addition, the system has supporting data analysis software, and with the help of the supporting software, data analysis becomes more convenient and fast. The system has been tested many times in practical applications, which greatly simplifies the operation process of the cone penetration test. Combined with the data analysis software, experimenters can obtain accurate soil properties at the test site. The experiment time is greatly shortened, and the durability of the new probe is better than the traditional probe, which reduces the maintenance cost of the equipment.

## Figures and Tables

**Table 1:** Reference for selection of strain gauges.

Series	Strain Limit	Fatigue Life	Main Application
EP	±10%	±1000/10^4^	Mainly used to measure large yield strain.
ED	±2%	±2500/10^6^	Suitable for dynamic measurement, not commonly used for static measurement.
BF	±3%	±1500/10^6^	Recommended for high-precision sensors.
WD	±2%	±2200/10^8^	Widely used in dynamic strain measurement.
WA	±2%	±2000/10^5^	Suitable for a wider temperature range and more extreme environment.

**Table 2:** Experimental data.

Time	Data1	Data2	Round
00:00:00	1016	2267	00007
00:00:15	1125	2513	00028
00:00:30	1382	2981	00047
00:00:45	1391	3012	00069
00:01:00	1278	2615	00085
00:01:15	1266	2578	00107
00:01:30	1184	2618	00125
00:01:45	1425	3075	00146
00:02:00	1276	2607	00163
00:02:15	1495	3196	00186
00:02:30	1469	3124	00207
00:02:45	1017	2285	00223
00:03:00	2673	3976	00241
00:03:15	2489	3583	00265

## Data Availability

No new data were created or analyzed in this study. Data sharing is not applicable.
